# Supplementary material for: The effect of metal remediation on the virulence and antimicrobial resistance of the opportunistic pathogen Pseudomonas aeruginosa
Source: Evol Appl. 2023 Jul 10;16(7):1377–89. doi: 10.1111/eva.13576 (PMC10363854; doi:10.1111/eva.13576)
Supplement: Supplementary file 1 — Data S1. [file EVA-16-1377-s001.docx]

Supplementary information for: The effect of metal remediation on the virulence and antimicrobial resistance of the opportunistic pathogen *Pseudomonas aeruginosa*

Running title: Liming and bacterial pathogenicity

Keywords: liming, antibiotic resistance, metal pollution, siderophores, *Pseudomonas aeruginosa*, opportunistic pathogen

This supplementary information contains plots of the growth curves used in the copper tolerance assay and of the survival probability of *Galleria mellonella* injected with the microbial community taken from the non-*Pseudomonas aeruginosa* inoculated samples.


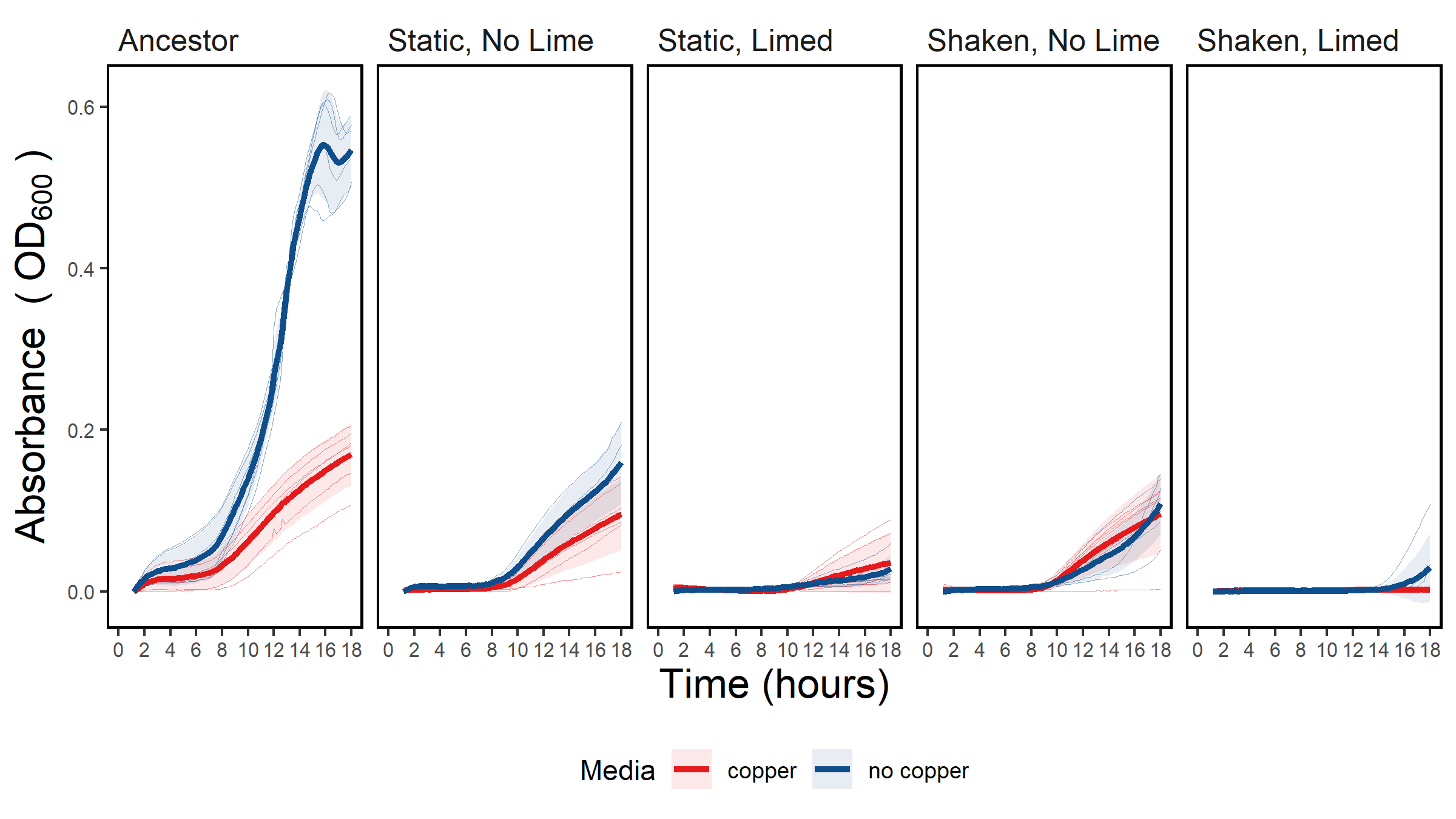


**Figure S1** The 18-hour growth curves of *Pseudomonas aeruginosa* populations incubated in either plain Iso-Sensitest broth (blue lines) or Iso-Sensitest broth containing toxic copper (1 g/L copper sulphate; red lines). The left panel shows the growth of the ancestral strain, and the other four panels show populations incubated in microcosms containing river water and sediment for 14 days with and without both lime and shaking. Thick lines show treatment means, thin lines the individual replicates, and shaded areas the 95% confidence intervals. We note that one replicate in the shaken, no lime treatment had a jagged growth curve and was consequently removed from the analysis (supplementary figure 2)


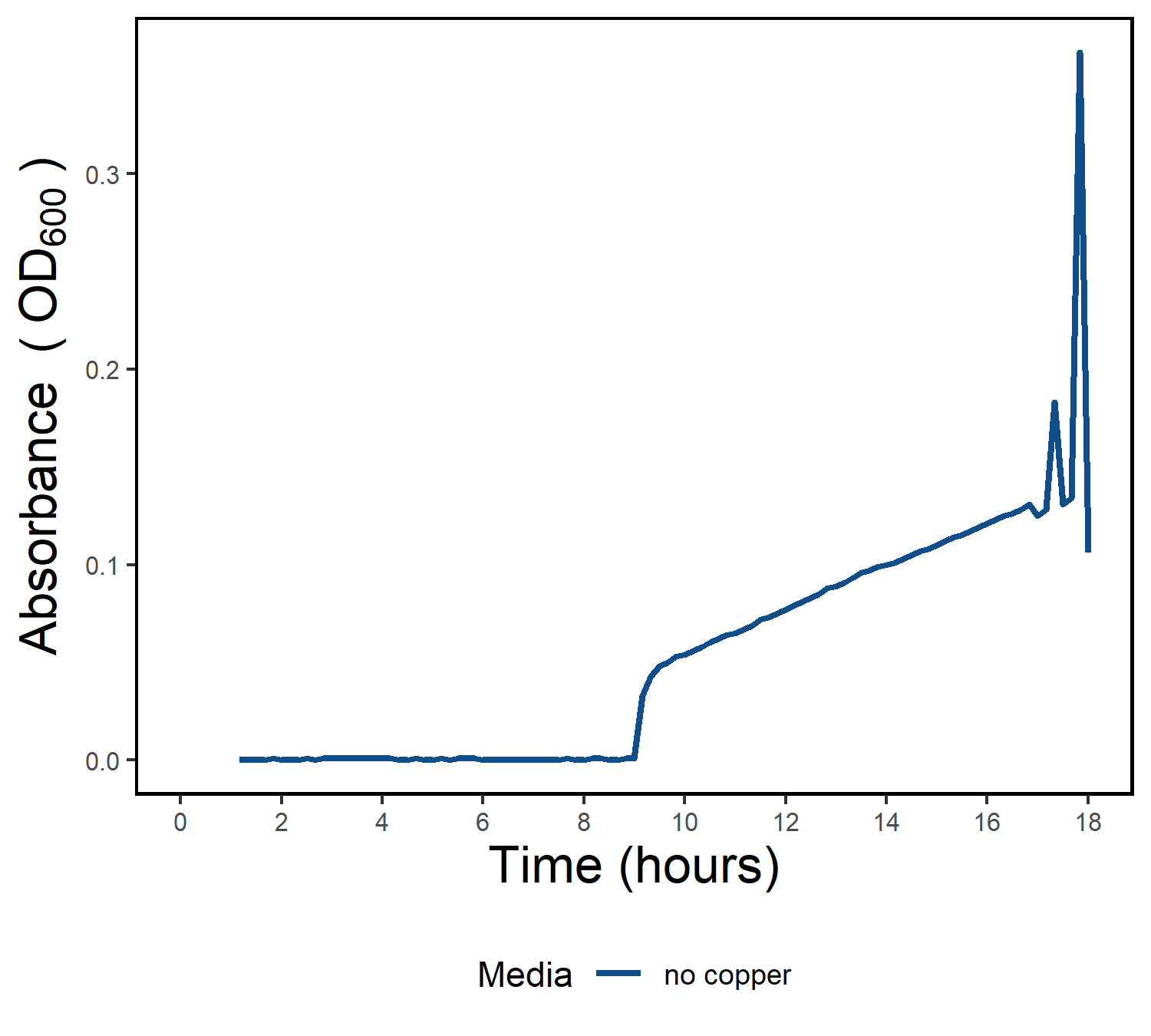


**Figure S2** The 18-hour growth curve of a *Pseudomonas aeruginosa* population incubated in plain Iso-Sensitest broth after being incubated in river water and sediment for 14 days in shaking and limed conditions. This replicate was removed from analyses due to the large fluctuations in OD after 16 hours.

**
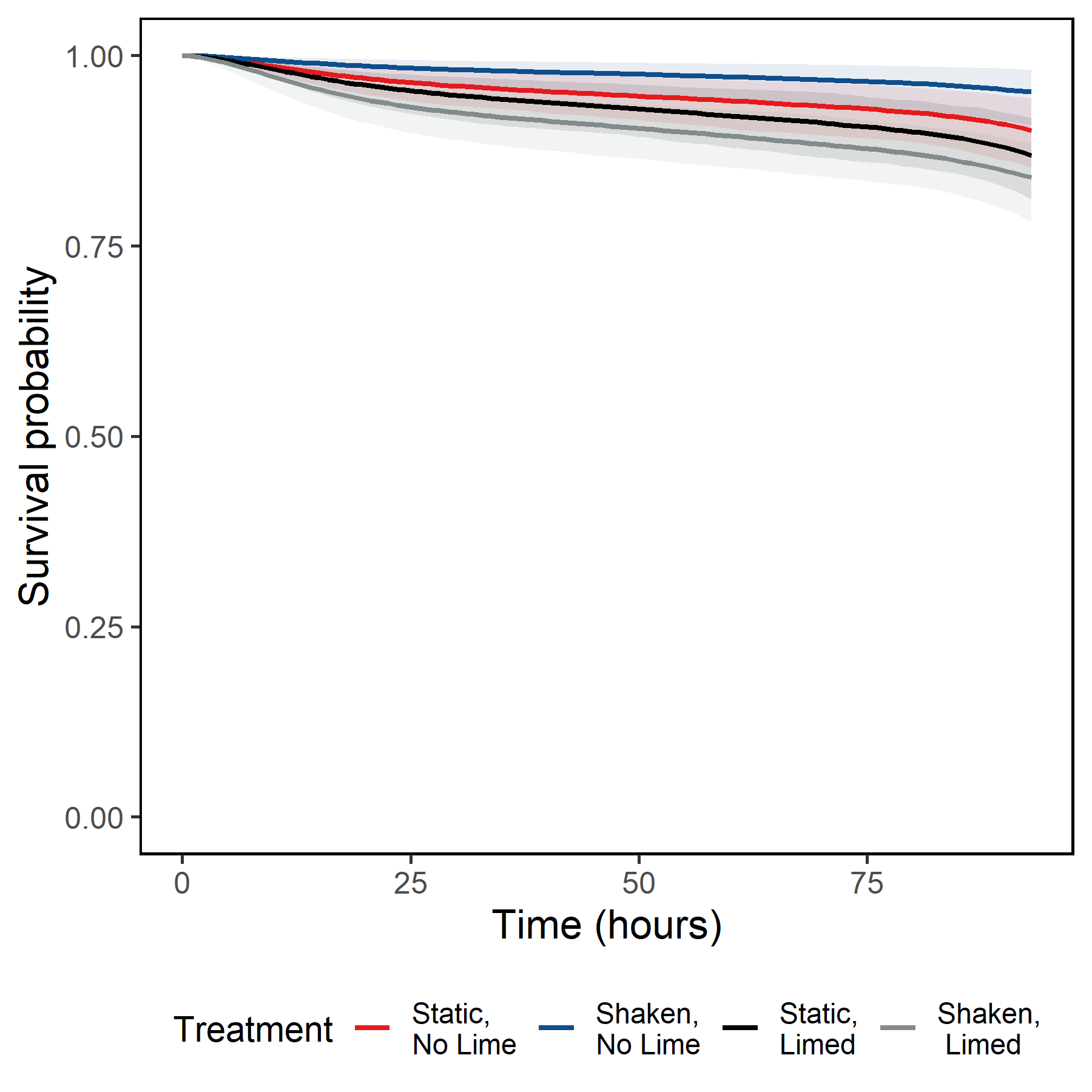
**

**Figure S3** The virulence (change in survival probability of 20 *Galleria mellonella* larvae per replicate) of the natural microbial community incubated in metal-contaminated river water and sediment for 28 days. Colours represent different treatments: grey = static, no lime; blue = static, limed; black = shaken, no lime; and red = shaken, limed. These do not significantly differ from one another. Shaded areas represent 95% confidence intervals.
